# Barriers and facilitators perceived by professionals while implementing Soothing and Sleeping in Dutch Healthcare: a mixed-methods study

**DOI:** 10.1136/bmjpo-2025-003478

**Published:** 2025-09-25

**Authors:** Jamie Lee van Someren, Eline Louise Möller, Hesther Rozemarijn Rodenburg, Annemieke E J Peters, Mariëtte H H Hoogsteder, Margreet W Harskamp-van Ginkel

**Affiliations:** 1 Quality of Care, Amsterdam Public Health Research Institute, Amsterdam, The Netherlands; 2 Public and Occupational Health, Amsterdam UMC, location Vrije Universiteit Amsterdam, Amsterdam, the Netherlands; 3 Amsterdam Reproduction & Development Research Institute, Amsterdam, The Netherlands; 4 Rehabilitation Medicine, Amsterdam UMC, location University of Amsterdam, Amsterdam, the Netherlands; 5 Stichting Esdege-Reigersdaal, Heerhugowaard, The Netherlands; 6 Department of Health Promotion, NUTRIM School of Nutrition and Translational Research in Metabolism, and Care and Public Health Research Institute, Maastricht University, Maastricht, The Netherlands; 7 Public and Occupational Health, Amsterdam UMC, location University of Amsterdam, Amsterdam, the Netherlands

**Keywords:** Nursing, Qualitative research, Sleep, Infant, Child Health

## Abstract

**Background:**

Many parents struggle with infant crying and sleep, yet some needs remain unmet by current professional support. Soothing and Sleeping, an evidence-based practice (EBP) based on the Happiest Baby method, offers preventive support and parental education for these issues. The Dutch Youth Health Care (YHC) system, monitoring 95% of children, is well-positioned to implement it.

**Method:**

This study explores YHC professionals’ perceptions of EBPs and Soothing and Sleeping and identifies key implementation barriers and facilitators. YHC professionals (n=41) completed questionnaires and participated in focus groups. Quantitative data were analysed using descriptive statistics and qualitative data through thematic analysis guided by the Dynamic Adaptation Process framework.

**Results:**

Professionals expressed positive attitudes towards EBPs and Soothing and Sleeping, with 81% adopting the method. Barriers and facilitators emerged at system, organisation, provider and client levels. Preimplementation, barriers spanned all levels, while postimplementation barriers were primarily systemic and organisational. Key barriers included resource limitations, inadequate training, organisational shifts and insufficient managerial support. Soothing and Sleeping-specific facilitators included its practicality, suitability and effectiveness.

**Conclusions:**

Findings suggest Soothing and Sleeping strengthens YHC support for infant crying and sleep. Recommendations include proactive parental outreach, appointing an implementation facilitator, ongoing training and tailored approaches for families.

**WHAT IS ALREADY KNOWN ON THIS TOPIC**

- Persistent infant crying and sleep problems are common in early childhood and are strongly associated with parental stress, burnout and strained parent–infant relationships.
- International research demonstrates that evidence-based soothing interventions can improve infant outcomes and enhance parental competence.
- Despite the potential benefits, the integration of such interventions into preventive child healthcare systems remains limited, and little is known about how professionals perceive their adoption in practice.

**WHAT THIS STUDY ADDS**

- New insights into Dutch Youth Health Care (YHC) professionals’ perceptions of Soothing and Sleeping as an evidence-based practice.
- Identification of system, organisational, provider and client-level barriers and facilitators to implementation, using the Dynamic Adaptation Process framework.

**HOW THIS STUDY MIGHT AFFECT RESEARCH, PRACTICE OR POLICY**

- Demonstrate the potential of Soothing and Sleeping to enhance parental support in managing infant crying and sleep problems.
- Provide practical strategies for effective implementation of Soothing and Sleeping in routine YHC practice.
- Contribute to policy and public health efforts aimed at strengthening parent-infant relationships, improving child well-being and reducing unnecessary healthcare utilisation.

## Introduction

 Many parents face unmet needs regarding infant crying and sleep problems, and infant healthcare is characterised by a high number of (unnecessary) referrals and hospitalisations.^1^,^3^ Parental support and education for excessive infant crying and sleep problems could be accommodated by Soothing and Sleeping, an evidence-based practice (EBP) based on the Happiest Baby Method.^4^,^6^ Recent studies have demonstrated its effectiveness in reducing (excessive) crying, improving infant sleep, decreasing feeding frequency and enhancing perceived maternal attachment and parental competence during the first year of life.^5^,^12^ The Happiest Baby Method is used by professionals and parents worldwide, but has not yet been implemented in the Netherlands.

Persistent infant crying can trigger parental burnout due to a cycle of constant demands and sleep deprivation.^13^ Parental exhaustion impairs nurturing behaviours, thereby intensifying infant distress and perpetuating crying. This negative cycle strains the parent–infant bond and heightens risks for postpartum depression, unsafe sleep practices and infant maltreatment.^114^,^16^ Integrating Soothing and Sleeping into current healthcare practices for (excessive) crying could help break this cycle. Soothing and Sleeping educates parents on the intrauterine effect or ‘missing fourth trimester’, infant crying and sleep, responsive cued care and the 5s’s: swaddling, side-stomach positioning, shushing, swinging and sucking.^4 17^ These steps may activate the infant’s calming response by mimicking soothing womb-like rhythms, thereby promoting self-regulation and sleep.^4 5 7^ Soothing and Sleeping enhances parental self-efficacy while fostering infant well-being.^8 18 19^

In the Netherlands, Youth Health Care (YHC) would be ideal for implementing Soothing and Sleeping. Dutch YHC is a preventive, community-based service delivered to all children (aged 0–18) and their families free-of-charge. They offer all vaccinations from the national immunisation programme, and regularly monitor children’s physical and mental health, they currently monitor 95% of all Dutch children.^20^ YHC nurses start with at-home and in-clinic visits 2 weeks after birth, when newborn crying patterns typically begin to increase, and together with medical doctors they keep monitoring the child regularly in community centres during the first years of life.^21 22^ YHC professionals are trained to observe parental responses to newborn’s needs, identify protective and risk factors for child development and recognise early signs of parental burn-out.^20^ They can provide adaptive extra support where indicated, for example, ruling out underlying medical causes for infant crying and implementing Soothing and Sleeping. Implementing Soothing and Sleeping in YHC aligns with current public health priorities to provide a solid start in life for every child, particularly those born in families in vulnerable situations.^23^,^25^

Integrating Soothing and Sleeping into current YHC practices has the potential to improve infant outcomes, but successful implementation may require local adaptations.^26^ The Dynamic Adaptation Process (DAP) Framework underscores how system, organisational, provider and client factors shape implementation.^26 27^ Maintaining fidelity while adapting methods is challenging, as professionals and organisation vary in practice despite standardised training.^27 28^ For instance, Dutch YHC organisations developed distinct local protocols for national obesity prevention guidelines, highlighting contextual influences on fidelity.^21^ Similarly, autonomous healthcare professionals tailor practices to client needs and may not immediately recognise new intervention’s benefits.^29^ Positive perceptions of an intervention are crucial for adoption.^28^

Understanding YHC professionals’ perceptions of EBPs and Soothing and Sleeping, along with implementation barriers and facilitators, is crucial to develop effective implementation strategies. This study examines these factors at system, organisational, provider and client levels in two Dutch YHC organisations.

## Methods

### Study design

This study was part of a larger study aimed at adapting Soothing and Sleeping to address the needs of families in vulnerable situations and evaluating the implementation process in Dutch YHC.^30^ Vulnerability in a family was defined as having a low income (defined as <150% minimum income), parental unfamiliarity with the Dutch healthcare system or long-term parental mental/cognitive vulnerabilities (ie, anxiety disorder, depression, AD(H)D, autism, low literacy, mild intellectual disabilities or substance abuse). The Medical Ethical Committee of Amsterdam UMC exempted the study from extensive review (2022.0208).

This mixed-methods study combined questionnaires and focus groups with YHC professionals (June 2022–April 2024). Qualitative data are the primary focus, with quantitative data complementing findings. This design strengthened the study’s findings and conclusions,^31^ allowing us to not only identify implementation barriers and facilitators but also explore underlying causes, informing recommendations for improvement.

### Participants

**Figure 1:**
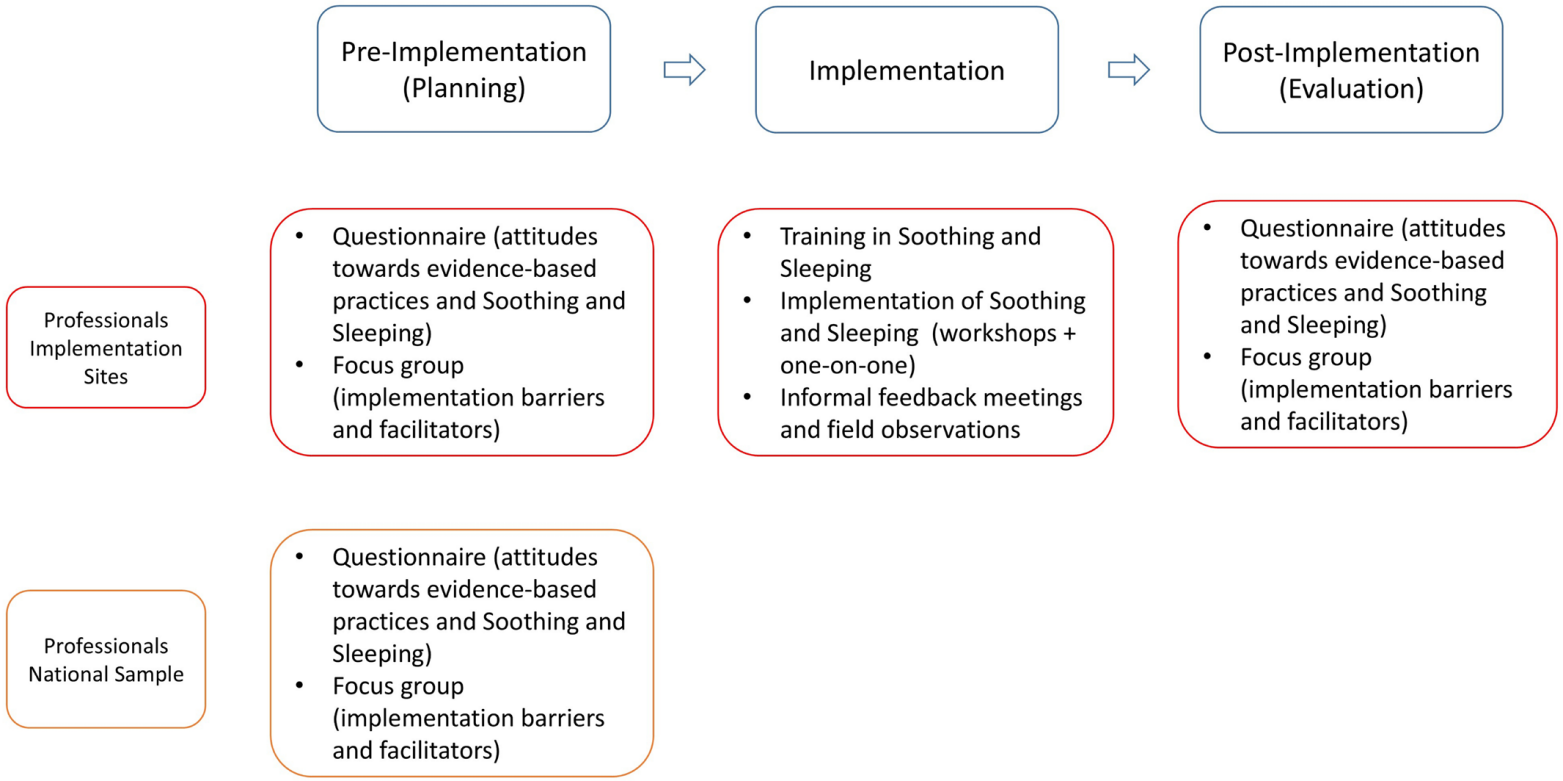
Visualisation of the implementation process and accompanying data collection methods for each phase and each participant group.

We observed Soothing and Sleeping implementation at two YHC centres (in Amsterdam and Alkmaar) across three phases: preimplementation, implementation and postimplementation (see figure 1). These sites are situated in neighbourhoods with high rates of low income, unemployment, addiction, chronic health issues and low education. To assess implementation site representativeness and gather insights on Soothing and Sleeping scale-up, we also involved a national sample of YHC professionals via the Dutch Center of Youth Health Care (NCJ).^32^

### Recruitment

All YHC professionals (youth nurses, medical doctors, policymakers and social workers) working at the implementation sites were recruited via their supervisors, while the NCJ invited national participants purposively based on expertise, location and role. The national sample included professionals trained to provide clinical and scientific feedback.^32^ All participants received written study information and provided written informed consent. Participation occurred during working hours or with hourly compensation.

### Data collection

Quantitative data were collected via questionnaires, and qualitative data through focus groups, as well as researcher field notes during site visits. This approach captured detailed experiences and emerging issues.

### Preimplementation phase

Participants completed an anonymous, digital questionnaire. The Evidence-Based Practice Attitude Scale (EBPAS-15) assessed attitudes towards EBPs,^33 34^ while selected determinants of the Measurement Instrument for Determinants of Innovations (MIDI)^35^ measured attitudes towards Soothing and Sleeping (online supplemental appendix AA). MIDI items were selected based on their relevance^1^,^8^ demographic variables included age, gender, role and experience. Semistructured focus groups (on-site/online) explored potential implementation barriers and facilitators. National sample focus groups additionally addressed Soothing and Sleeping scale-up. Focus groups (37–64 min) were led by two facilitators (JLvS, MHHH, MWH-vG, ELM and trained facilitators), audio-recorded and guided by detailed topic guides (online supplemental appendix B).

### Implementation phase

Implementation involved: (1) team training in Soothing and Sleeping and (2) integrating Soothing and Sleeping into practice by professionals through individual parental guidance or parental workshops (see online supplemental appendix C). During the implementation phase, researchers (JLvS, MHHH, AEJP) conducted informal site visits, engaged with professionals and observed Soothing and Sleeping consultations or workshops, while taking field notes. For parental workshops, families were charged €5 if they had a low income and €25 otherwise. The fee covered the provision of a swaddle blanket and an informational booklet for home use. The fee also helped, based on the organisation’s prior experience, to reduce non-attendance, as free registration was associated with higher rates of no-shows.

### Postimplementation phase

Implementation site professionals’ attitudes were reassessed using the EBPAS-15 and determinants from the MIDI (see online supplemental appendix A). Focus groups (81–105 min) explored implementation barriers and facilitators. Two facilitators (JLvS, ELM, AEJP) led focus groups, following structured guides (online supplemental appendix B).

### Quantitative analysis

Questionnaire data were analysed using IBM SPSS Statistics 28, employing descriptive statistics to compare implementation sites and the national sample. Preimplementation and postimplementation responses were compared based on mean scores, to assess attitude shifts attributable to study participation. We did not test for statistical significance, due to the small sample size and exploratory nature of the quantitative analysis, neither did we identify any norms suitable to compare our EBPAS-15 or MIDI scores to.

### Qualitative analysis

Focus groups were transcribed verbatim and coded using MAXQDA V.2020.^36^ Analysis followed the DAP framework,^26^ addressing system, organisation, provider and client levels, with additional inductive coding.^37^ Regarding the DAP framework, our focus for this paper is on professionals’ views on the four levels, and we are not reporting on clients’ own views or perceptions. For each phase, two researchers (JLvS and MHa) double-coded two transcripts independently, resolving discrepancies collaboratively and updating the codebook. High agreement allowed JLvS to code remaining transcripts independently. Thematic analysis^37^ categorised codes by DAP framework level and clustered these into overarching themes, which were reviewed by the research team. To enhance transparency and credibility, we reflected on how the professional and personal backgrounds of the research team may have influenced data collection, facilitation and interpretation in online supplemental appendix D. To further enhance credibility, participants were invited to review and provide feedback on the preliminary findings during two separate meetings. These findings were also presented to a broader audience at two annual local meetings attended by professionals, researchers and policy makers involved in YHC. In addition, preliminary results were regularly discussed in project group meetings comprising one participant, researchers and policymakers. All feedback was considered during interpretation of the results.

### Patient and public involvement

In this study, we had regular meetings with an advisory board, including future users of Soothing and Sleeping (professionals in youth healthcare) and policymakers involved in early infant healthcare. Moreover, in the overarching study, clients (eg, parents) were consulted on recruitment of participants for the study, for the workshops and other matters such as workshop materials. Currently, we are developing educational videos on the Soothing and Sleeping method in cocreation processes with parents and professionals.

## Results

### Participant characteristics

In total, 41 YHC professionals participated in this study, including 16 from the national sample and 25 from the implementation sites. For the implementation sites, all invited team members participated preimplementation, but participation throughout the implementation process was influenced by staff turnover, sickness and maternity leave. Therefore, 12 participants contributed across all phases, 7 participated only before and during implementation and 6 during and postimplementation (they were included at a later stage). For the national sample, 16 participants were selected and agreed to participate from a group of 40 active members of an YHC science consultant group. Professionals at the implementation sites were younger and had less work experience than those in the national sample (see table 1).

**Table 1:** Demographic characteristics of participating healthcare professionals

Variable	National sample (n=16) preimplementation	Implementation sites (n=25) preimplementation and postimplementation
	%	%
Female gender	100	96
Position		
Youth nurse	50	75
Medical doctor	44	17
Project leader	6	0
Social worker	0	8
Age		
<30	6	29
30–39	19	25
40–49	31	21
>50	44	25
Years of experience		
<5	25	50
6–10	0	17
11–14	19	0
>15	56	33

### Professional’s attitudes towards EBPs and Soothing and Sleeping

**Figure 2:**
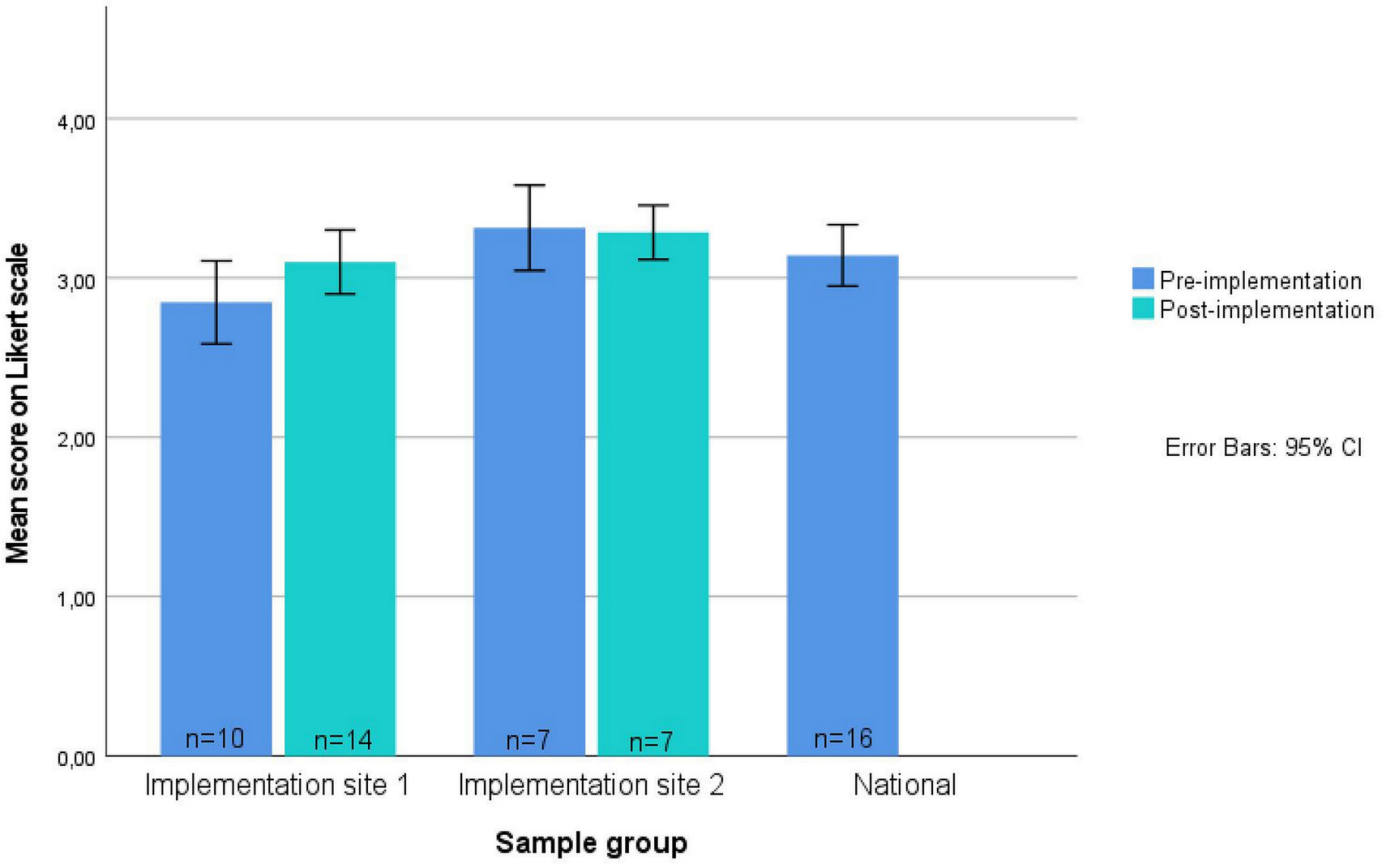
Bar graph of mean scores on the EBPAS-15 by group and implementation phase (total n=41). The 5-point Likert scale ranged from 0 to 4, with higher scores indicating a more positive attitude. EBPAS-15, Evidence-Based Practice Attitude Scale.

Professionals at the implementation sites showed positive attitudes towards EBPs preimplementation, comparable to the national sample, with no significant differences between groups. The total mean EBPAS score for the implementation sites appeared higher postimplementation (M=3.64, SD=0.32) compared with preimplementation (M=3.04, SD=0.31), due to a slight rise in score in implementation site 1, while scores in implementation site 2 remained similar (see figure 2).

Preimplementation MIDI results indicated that professionals felt responsible for using Soothing and Sleeping, considered it relevant for parents and anticipated high client satisfaction, with no changes postimplementation (data not shown). The national sample scored lower on relevance and expected client satisfaction. Initially, professionals at both implementation sites reported minimal influence from the expectations of colleagues, supervisors and parents regarding Soothing and Sleeping use, but felt slightly more affected postimplementation.

In the postimplementation questionnaire, 48% of professionals reported skimming through Soothing and Sleeping training materials, while 52% read them thoroughly. Overall, 81% integrated Soothing and Sleeping into their daily practice, either by leading Soothing and Sleeping workshops, applying it during individual in-clinic or home visits, referring parents to workshops or colleagues or using parts of the method. Professionals who did not use the method either stopped working with the target group or had not encountered a need to apply it.

For the EBPAS, the relatively high scores preimplementation may partly explain why only limited change was observed postimplementation. Nevertheless, the modest increase at one site suggests a further strengthening of positive attitudes after implementing Soothing and Sleeping, while stable scores at the second site suggest sustained receptivity. For the MIDI, the fact that professionals already perceived Soothing and Sleeping as relevant and expected high client satisfaction may also explain the lack of measurable change postimplementation. However, the slight increase in reported influence from colleagues, supervisors and parents suggests that organisational and social dynamics became more important during implementation. Moreover, the high rate of integration into practice (81%) underscores the intervention’s feasibility and acceptability in YHC.

### Experienced barriers and facilitators for implementation

Various barriers and facilitators were identified. Because they often overlapped, we decided to discuss the results in themes, rather than listing barriers and facilitators separately. Nine themes emerged from thematic analysis of all focus groups, summarised in table 2, and categorised by the DAP framework levels: system, organisation, provider and client.^26^

**Table 2:** Overview of themes and subthemes identified in all focus groups (preimplementation: n=3 national sample, n=3 implementation sites and postimplementation: n=3 implementation sites), categorised by DAP framework levels

Level	Preimplementation theme and subthemes	Postimplementation theme and subthemes
System level	Resources and organisational shifts	
	Healthcare staff shortages (−)* Time constraints (−) National task reallocations (−) YHC focusing less on prevention (−)	Healthcare staff shortages (−) Time constraints (−) National task reallocations (−) YHC focusing less on prevention (−) Early explanation of Soothing and Sleeping saves time at later stage (+)
	Parental needs and (mis)perceptions of YHC	
	Identified parental need for support with infant crying/sleep (+) Soothing and Sleeping fits parental needs (+) Unawareness of YHC expertise (−) Misperceptions about YHC (−)	Soothing and Sleeping suits parental needs for support (+) Unawareness of YHC expertise (−) Misperceptions about YHC (−)
Organisation level	Role management/organisation	
	Mismatch between expectations pilot team and management (−)	Insufficient compensation for extra time spent on implementation (−) Organisation provided extra location for workshops (+) Minimal focus on Soothing and Sleeping in team/organisation meetings (−) No registration of crying and intervention in patient files (−) Refreshment courses needed (−)
	Accessibility to Soothing and Sleeping for families in vulnerable situations	
	YHC reaches families in vulnerable situations with more difficulty (−)	Time constraints limit possibilities for necessary individual explanation (−) Organisation provided workshops to some families without costs (+) Families did not follow-up referrals by professionals for unclear reasons (−) Attendance for other weekly walk-in interventions is high for all families (+)
Provider level	Standardisation of practice	
	Professionals relying on own experience (−) Uniformity would be beneficial (+) Worries about Soothing and Sleeping replacing other methods (−) Worries about use Soothing and Sleeping as quick fix (−) Worries about personalised care with Soothing and Sleeping (−)	Method is a welcome and effective addition to help parents (+) Not used as quick fix—method can be tailored to individual families (+)
	Integrating Soothing and Sleeping into current practice	
		High provider satisfaction (+) If Soothing and Sleeping does not work, professionals still use other methods (+) Every childcare professional should know Soothing and Sleeping (+) Professionals perceive the costs workshop as a barrier (−) Costless registration more often led to cancellations and no-shows (−)
	Practicality	
	Practical steps (+)	Practicality of the method (+) Non-verbal explanation possible in case of a language barrier (+) Materials for parents are supportive (+) Some parents have reluctance towards swaddling (−)
	Training	
	Preparatory reading partly in non-native language (−)	Training insufficient without refreshment sessions (−) Practical skills that you need to maintain by repetition (−) Competing agenda (−) Training materials supportive to review (+)
Client level	Parental experiences	
		Positive feedback parents (+) Soothing and Sleeping reduces questions about crying at later stage as well (+) Professionals find Soothing and Sleeping suitable to support parents (+) High heterogeneity in motivation of parents to follow-up referrals (−)

* Plusses (+) indicate that professionals considered this to be a facilitator for implementation, whereas minuses (−) were considered barriers.

DAP, Dynamic Adaptation Process; YHC, Youth Health Care.

### System level

Two system-level themes were identified: (1) resources and organisational shifts and (2) parental needs and (mis)perceptions of YHC services.

#### Resources and organisational shifts

Preimplementation, professionals identified national healthcare staff shortage as a major barrier to Soothing and Sleeping implementation, citing high workloads and limited consultation time. With only 15 min per consultation to cover 30 items, explaining Soothing and Sleeping thoroughly seemed challenging: *To explain something very well and motivate people, you need reasonable time.* (p8, medical doctor, site 1).

To address staff shortages, YHC nationally reallocated tasks from medical doctors to nurses, increasing their outpatient clinic hours. However, this transition, alongside continued staff shortages, caused organisational turbulence, as reported by 71% of the professionals preimplementation and 45% postimplementation. Nurses also noted a broader shift in YHC’s focus, transitioning *from preventive parental education to question-oriented parental education and care.* (p4, youth nurse, site 1). This reduced focus on prevention was noticed at both implementation sites: *The organization may say prevention is number one but that’s not how I experience it anymore.* (p6, youth nurse, site 2). This reduced early intervention for excessive crying, only addressing it when *shit had hit the fan*.

Early introduction to Soothing and Sleeping through workshops, however, proved beneficial: *A lot is already explained at the workshop. If parents attend it before their three month consultation it saves us all those questions, and we're a step ahead of it.* (p6, youth nurse, implementation site 1).

For effective Soothing and Sleeping integration, professionals stressed that *it is crucial that it aligns with how YHC organizations already operate*. (p1, medical doctor, national sample). While postimplementation feedback indicated compatibility with current practice, time constraints continued to pose a barrier, potentially leading to superficial explanations in consultations: *This shortage of time may result in professionals explaining Soothing and Sleeping too fleetingly to parents, resulting in ineffectiveness at home*. (p2, youth nurse, site 1). To address time limitations, professionals recommended integrating Soothing and Sleeping into existing *Centering Parenting* groups^38^ or informal walk-in sessions.

#### Parental needs and (mis)perceptions of YHC

Before implementation, professionals identified a strong parental demand for support with infant crying and sleep problems. They noted that many parents today prefer quick solutions, making current guidelines less aligned with their expectations. Soothing and Sleeping was seen as a better fit:

Parents today think children shouldn’t cry, they find it difficult, and don't have the time or patience to work on it for a longer period. I think current practices don’t match parents anymore, so offering something very practical [like Soothing and Sleeping] would help. (P15, youth nurse, national sample)

Postimplementation, 95% of the professionals agreed that Soothing and Sleeping suited the parents they work with. However, a key barrier remained: many parents were unaware of YHC’s role in addressing infant crying and sleep issues, often seeking help too late:

We are almost always involved too late. Parents don’t think of us when problems start. They go to the GP or even a private sleep coach, whereas we could have stepped in earlier with accessible solutions, preventing escalation to more expensive care. (P6, medical doctor, national sample)

Professionals also emphasised the need to modernise YHC’s communication strategies to counter parental perceptions that it is *old-fashioned*. Additionally, some parents—particularly those hesitant to engage with professional care—mistake YHC for child protection services: *You could keep ringing the doorbell, but many are afraid to let authorities in, and they think we're one of them too.* (p5, youth nurse, site 1)

### Organisational level

Managerial and institutional support played a crucial role in Soothing and Sleeping implementation. While some support facilitated adoption, gaps in other areas posed barriers. Professionals also expressed concerns that families in vulnerable situations (eg, low education, low income, mental health issues, parental addiction) might struggle to access care.

#### Role of management/organisation

Preimplementation, some professionals felt excluded from decision-making processes by the organisation and management: *Lots of changes are happening, and we're not asked what we need to make it work.* (p1, youth nurse, site 2). However, the organisation facilitated implementation by addressing emergent barriers on professionals’ request, such as adding workshop locations closer to staff and parents.

Postimplementation, professionals noted limited team awareness of Soothing and Sleeping: *I think it works very well, but the topic has to be more alive within the team*. (p6, youth nurse, site 1). The MIDI questionnaire revealed mixed perceptions of Soothing and Sleeping adoption. Half of the professionals believed less than 50% of their trained colleagues used it, while the other half estimated adoption exceeded 50%. Limited team communication, possibly due individual in-clinic work was cited as a contributing factor: I *wanted to consult a colleague about the method, but we did not cross paths and then I forgot about it.* (p8, medical doctor, site 2).

To improve team awareness and enhance Soothing and Sleeping adoption, professionals suggested discussing it regularly in team meetings, offering refresher courses every 6 months, and integrating it into their registration system: *One downside is that Soothing and Sleeping is not included in our reporting system. A reminder for undiscussed topics would help increase awareness.* (p3, youth nurse, site 1).

### Accessibility to Soothing and Sleeping for families in vulnerable situations

Professionals had the opportunity to introduce Soothing and Sleeping to parents through in-clinic visits, home visits or workshops, but they anticipated challenges in motivating parents to attend group workshops, citing barriers such as transportation, childcare and education levels. One nurse highlighted additional challenges for families in vulnerable situations in accessing Soothing and Sleeping:

They're at their wit’s end, barely have any [head] space for that baby, let alone to reflect on it, and they often can't. I do foresee a number of problems. But that doesn't mean we shouldn't implement it. (p10, youth nurse, site 1)

Accordingly, home visits were seen as more suitable for these families than workshops, and some were reached this way. However, time constraints limited professionals’ ability to extend this service to all families who might benefit. To facilitate workshop attendance, the organisation allowed families in vulnerable situations to join workshops free of charge, yet some still did not attend for unclear reasons.

Both pre and postimplementation, professionals recommended proactive outreach strategies to lower participation barriers for parents in vulnerable situations, such as visiting locations already attended by these parents and collaborating with partner organisations. They further suggested maternity care nurses could introduce Soothing and Sleeping during early home visits and refer parents to YHC. Additionally, weekly walk-in hours on other topics proved effective in engaging families in vulnerable situations, demonstrating the value of integrating Soothing and Sleeping into existing services.

### Provider level

Before implementation, professionals anticipated challenges in integrating Soothing and Sleeping into practice and feared it might become a ‘quick fix’. However, postimplementation, they reported high satisfaction, finding that the method facilitated their work. Standardisation of practice was seen as both a facilitator and a challenge. While the method’s practicality was identified as a key facilitator, this also brought challenges such as the need for repeated training.

#### Standardisation of practice

Pre-implementation, professionals expressed concerns about adherence to new guidelines due to nurses’ reliance on experiential knowledge:

Guidelines aren’t always followed completely. There is commitment to practical experiences, with senior nurses passing down knowledge to younger nurses. (P1, medical doctor, national sample)

Moreover, professionals feared Soothing and Sleeping would replace their current approach, typically based on each family’s unique situation and support system. They warned against viewing it as a *one-size-fits-all solution* or *quick-fix*. On the other hand, they recognised that standardisation introduced by Soothing and Sleeping could facilitate care delivery:

Care offered to parents currently varies depending on the nurse. It would be nice to have one evidence-based method, making care more predictable for professionals and parents. (p16, medical doctor, national sample)

Concerns about Soothing and Sleeping replacing personalised care were alleviated postimplementation, as professionals found it adaptable to individual family needs:

You can explain it in detail or keep it practical. The hands-on approach works well for doers, you show them and they copy you, while others want more in-depth explanations. (p1, youth nurse, site 2)

#### Integrating Soothing and Sleeping into current practice

Professionals stated that rather than replacing existing practices, Soothing and Sleeping complemented medical check-ups or advice, aiding in distinguishing between temporary excessive crying and persistent parent–child interaction issues. It facilitated earlier identification of persistent issues—by prompting further assessment when crying or interaction problems persisted after deploying Soothing and Sleeping—and timely referrals to specialised care.

Postimplementation MIDI questionnaires indicated high provider satisfaction, with professionals reporting increased confidence in addressing crying and sleep issues, improved relationships with parents and enhanced father involvement. While not all trained professionals fully adopted Soothing and Sleeping, there was strong interest among all for its integration into routine practice: *We need all professionals trained so that we can promote excellent guidance for excessive crying within our organization.* (p6, youth nurse, site 2). Professionals also advocated for broader dissemination to GPs, hospitals and midwives to improve referrals to YHC, provided that they would have adequate staffing and time.

Finally, some professionals expressed concerns about workshop costs. Since most services at YHC are free-of-charge, they felt uncomfortable referring parents to workshops with associated fees. On the other hand, they noted that costless registration often led to higher no-show rates.

#### Practicality

Preimplementation, professionals anticipated Soothing and Sleeping’s practicality would be facilitating, providing concrete steps to parents rather than mere advice, which was confirmed postimplementation: *Previously, we just reassured parents about crying. Now we offer practical guidance. It feels good to be able to provide that to parents in need.* (P2, youth nurse, site 1). Practical demonstration also helped overcome language barriers, making the method widely applicable: *sometimes it works to just show them, this method is perfect for that, it is just five practical consecutive actions that they can imitate.* (p5, youth nurse, site 1).

Furthermore, the easy accessibility to materials necessary to use Soothing and Sleeping facilitated implementation: *Parents usually already have a blanket for the swaddling, so it’s easy to show them without getting materials.* (p4, youth nurse, implementation site 1). However, some parents were initially reluctant towards swaddling: *They say their child doesn't like swaddling when they’ve only tried it once. Then I explain them it’s scientifically proven to work, even for babies who initially don’t like it.* (p5, youth nurse, site 1)

#### Training

Preimplementation, concerns about English-language preparatory reading materials led partial translations, yet some professionals still did not review them. The 2-hour team training with preparatory reading was considered insufficient by some, particularly for mastering swaddling techniques:

Initially I found the swaddling quite difficult. You really need experience to be able to teach the swaddling steps to someone else. If you haven't mastered it yourself, it’s just very difficult. (p3, youth nurse, site 2)

Interestingly, the postimplementation MIDI questionnaire revealed that most professionals felt comfortable explaining Soothing and Sleeping to parents, but consistent practice was essential to retain knowledge: *A barrier is losing knowledge if you don’t repeatedly use it.* (p2, youth nurse, site 2).

Without consistent use, Soothing and Sleeping risks becoming deprioritised among professionals’ competing demands: *where it becomes one of so many things you have to do*. (p2, youth nurse, implementation site 2). This was also observed during informal visits by the researchers: while professionals proposed organising refresher courses or swaddling practice sessions, these initiatives never followed through. However, written training materials and instructional videos helped reinforce learning: *just to remind me of how it’s done again.* (p19, youth nurse, site 1). One nurse, who actively sought peer guidance and practice, was able to integrate it effectively into her daily practice, even after initially only receiving the 2-hour training session with preparatory reading.

By assisting with workshops, and applying what I learned, I noticed the knowledge stuck. Now I can pass on the theoretical background and practical steps I’ve learned from my colleagues and at the training to parents during consultations, so that parents don’t have to wait until the workshop. It is embedded in my routine now. (p12, youth nurse, site 1)

### Client level

#### Parental experiences

Postimplementation, professionals reported receiving consistently positive parental feedback on Soothing and Sleeping:

When parents use the steps correctly and understand why they do it, Soothing and Sleeping always works. In workshops, most babies are asleep by the end. During in-clinic check-ups later on, they are doing well and parents rarely have questions about crying anymore. (p3, youth nurse, site 2)

Soothing and Sleeping’s theoretical background helped parents understand their infant’s needs and accept their high need for soothing. Professionals indicated in the MIDI postimplementation that Soothing and Sleeping enhanced tailored care, increased parental confidence in responding sensitively to their baby, offered practical advice on sleep issues and enabled parents to learn about infant care without requiring them to read extensive materials.

However, follow-through on professional referrals varied widely between families. Active follow-up, such as reminder calls, improved participation, but reasons for non-compliance remained unclear.

## Discussion

### Main findings

This study investigated YHC professionals’ perceptions of EBPs and Soothing and Sleeping and identified barriers and facilitators to implementation. Both preimplementation and postimplementation, professionals expressed positive attitudes towards EBPs and Soothing and Sleeping, a key facilitator for successful implementation.^28^ For the EBPAS, the relatively high score preimplementation may partly explain why only limited change was observed postimplementation. Nevertheless, the modest increase at one site suggests a further strengthening of positive attitudes after implementing Soothing and Sleeping, while stable scores at the second site suggest sustained receptivity. For the MIDI, the fact that professionals already perceived Soothing and Sleeping as relevant and expected high client satisfaction may also explain the lack of measurable change postimplementation. However, the slight increase in reported influence from colleagues, supervisors and parents suggests that organisational and social dynamics became more important during implementation. Moreover, the high rate of integration into practice (81%) underscores the intervention’s feasibility and acceptability in YHC.

However, preimplementation focus groups revealed initial scepticism, with professionals anticipating barriers at system, organisation, provider and client levels. General barriers included resource constraints and accessibility issues for families in vulnerable situations, while Soothing and Sleeping-specific concerns involved fears of it becoming a ‘quick-fix’ and parental misperceptions of YHC. Anticipated facilitators were largely Soothing and Sleeping-specific, such as its practicality, suitability for parents and potential to standardise care.

Postimplementation, many anticipated barriers were alleviated, especially on the provider and client level. Psychological reactance theory may explain initial scepticism, as professionals may have perceived the method as a threat to autonomy.^39^ They later reported high satisfaction with Soothing and Sleeping’s impact, ease of integration and adaptability to parental needs, including for families in vulnerable situations. However, accessibility for these families remained challenging due to the increased time investment required from professionals. While professionals supported integrating Soothing and Sleeping into standard care, system-level and organisation-level barriers persisted, including resource constraints, insufficient managerial support and insufficient training. Notably, these barriers align with broader healthcare implementation challenges.^40^,^44^ Moreover, external barriers like these are found to be more readily discussed by professionals than internal factors like motivation, as they fall outside their direct control.^41 43 45^

To our knowledge, this study is the first to explore Soothing and Sleeping implementation in YHC. While prior research evaluated its effectiveness in hospitals and home visits, those studies primarily involved researcher-led parental education rather than healthcare professionals in routine practice.^9 11 12 46^

### Implementation fidelity challenges

Incomplete adoption of Soothing and Sleeping in this study could be attributed to healthcare professionals’ tendency to adapt practices to individual client needs and high autonomy.^21 29 40^ This autonomy allows them to independently decide whether and how extensively to adopt new practices. Additional context-specific factors may have also played a role.

The preparatory materials, read thoroughly by only half of the professionals, may have hindered full implementation. This insufficient preparation may be attributed to language barriers, as some content was in professionals’ non-native language, advocating for the development of Dutch-language training materials. Limited preparation, together with the 2-hour team training, led some to perceive the training as inadequate for mastering Soothing and Sleeping. This may have led to partial implementation, such as focusing on swaddling or shushing only, despite evidence that simultaneous use of all five steps is critical for its effectiveness.^47^ A thorough understanding of the rationale behind the steps is, therefore, crucial for successful integration into daily practice.

Limited training is a known barrier to implementation and may also reduce confidence in applying the method, as seen in previous community nursing studies.^40^ The tendency of many professionals in this study to refer parents to workshops or more extensively trained colleagues supports this possibility. Nevertheless, the majority of professionals reported confidence in explaining the practical steps of the method in the postimplementation questionnaire. Therefore, other factors such as time constraints may play a bigger role than reduced confidence.

Limited time to learn and master new skills—such as swaddling—is a known obstacle to adoption of innovations.^44^ Professionals recommended longer training support and periodic refresher courses, which align with previous research.^40^ In our study, nurses with extended training and Soothing and Sleeping certification also demonstrated better implementation. Online supplemental materials like booklets and instructional videos were valued, aligning with other implementation studies.^40 48^

Finally, the independent nature of YHC professionals’ work limits peer exchange and team awareness about new interventions. Implementing ‘local champions’ could foster collaboration, awareness and improve sustainability of new interventions.^40 49^ Nurses at our pilot sites did not use the knowledge of Soothing and Sleeping educators in their team—who could have functioned as local champions—for this purpose. Regular management-initiated meetings or champion-led sessions could further enhance team engagement and address implementation barriers.^40^

### Contextual influences on implementation: organisational shifts and support by management

During the research period, task reallocations were ongoing at both the implementation sites and nationally, with nurses taking on additional in-clinic visits and medical doctors gaining time for interdisciplinary collaboration.^50 51^ However, in practice, Soothing and Sleeping-certified nurses primarily led interdisciplinary collaboration efforts. Meanwhile, Dutch policymakers increased focus on Early Life Stress and initiatives that support infants’ early development.^25^ Temporary funding allowed professionals to raise awareness for Adverse Life Events and increase perinatal interdisciplinary collaborations and setting up new interventions like Soothing and Sleeping, but lacked sustainable budget increases to match inflation.^25^ As professionals noted, as a publicly funded organisation, YHC remains vulnerable to shifting political priorities. Funding determined by fixed fees rather than service volume limits professionals’ ability to promote their services and provide additional Soothing and Sleeping-related care.

Organisational turbulence, a known barrier to innovation adoption,^49^ may have contributed to dissatisfaction with managerial support and time constraints. Reorganisations often divert professionals’ time and energy from implementation of a new practice, and management may also prioritise organisational changes over new interventions.^49 52^ Experienced barriers in this study reflect these challenges. Future efforts should consider organisational stability and appoint site-based facilitators to oversee implementation, ensure team communication and train local champions, especially during turbulent times.^48 53 54^ In this study, researchers acted as such facilitators.

### Improving parental perceptions of YHC services to ensure accessibility to Soothing and Sleeping for all families

Professionals observed that parents often lacked awareness of YHC services for infant crying and sleep, which could hinder access to Soothing and Sleeping. Previous Dutch studies similarly highlight confusion around YHC services and negative parental perceptions.^55 56^ To reach all parents with Soothing and Sleeping, it is essential to inform parents about the free expertise offered by YHC. Over time, national efforts should aim to reshape the public image of YHC to avoid parental misperceptions.

To further improve accessibility to Soothing and Sleeping, proactive outreach and tailored approaches for families in vulnerable situations are crucial. These families were reached well by individual consultations but to a lesser degree with Soothing and Sleeping group workshops. The physical, mental and emotional barriers they may face in attending group workshops, such as transportation, childcare, education levels and stress, could make one-on-one care more suitable.^56^ On the other hand, studies among mothers in Dutch YHC show that group-based interventions, even for mothers in vulnerable situations, have substantial benefits such as learning through interaction, normalisation and building social networks.^57 58^

To facilitate participation in existing or new (group-based) interventions, collaborating with intermediaries or informal community leaders is known to be effective.^56 59 60^ Intermediaries can enhance intercultural competencies, clearly communicate available services and facilitate culturally sensitive care. Engaging families in vulnerable situations—where excessive crying is more often present—and educating them about Soothing and Sleeping early in a newborn’s life is essential for maximising benefits to infants, parents and society.^25^

### Strengths and limitations

A key strength of this study was its mixed-methods approach, with multiple measurement points to capture professionals’ experiences before, during and after implementation of Soothing and Sleeping. Additionally, we used two settings—a larger city and smaller one—to pilot Soothing and Sleeping, both of which showed similar openness to EBPs as the national sample, suggesting representativeness on this aspect. Finally, the predominance of female participants in our sample reflects the gender distribution within the Dutch YHC field.^61^

A limitation of this study is the partial adoption of Soothing and Sleeping by some professionals, which may have influenced perceptions of its effectiveness and experienced implementation barriers and facilitators. Additionally, the research team provided more support than would typically be available in real-world settings, which could mean certain barriers and facilitators are stronger in future implementation efforts. Another limitation is that the cultural and linguistic backgrounds of professionals were not assessed, although these factors may influence their acceptance of interventions such as Soothing and Sleeping, particularly components like swaddling. Swaddling is considered a common and beneficial soothing practice in some cultural contexts, whereas in others, it may be discouraged due to concerns about infant safety or developmental outcomes.^62^ Furthermore, differences in language and culture between professionals and families may affect how confidently recommendations are communicated and received.^63^ Such barriers could potentially influence both the uptake by professionals as well as by parents. However, our MIDI measures indicated that professionals considered Soothing and Sleeping relevant for parents, and 81% integrated Soothing and Sleeping into their daily practice, with professionals not implementing it providing only reasons such as not working with the target group or not encountering a need to implement it.

### Recommendations for future implementation

To ensure programme fidelity and effectiveness when scaling up Soothing and Sleeping implementation in YHC, it is crucial to plan implementation strategies accordingly. Recommended strategies are summarised here by DAP framework level.

- System level: actively inform parents about YHC’s free expertise, especially for families in vulnerable situations, through proactive outreach and community partnerships.
- Organisational level: appoint a facilitator to oversee implementation and improve communication between teams and management. Ensure organisational stability when introducing new practices.
- Provider level: ensure adequate preparation before training, skill practice and ongoing support post-training. Utilise local champions and online supplemental materials to reinforce learning.
- Client level: tailor care to individual family situations. Highlight practical steps. Offer group workshops for those without attendance barriers and one-on-one care or weekly walk-ins for those with these barriers.

### Future research

When implementation strategies are used, we recommend process evaluation to investigate fidelity and sustainability of Soothing and Sleeping across different care settings. Key areas for evaluation include: the impact of actively informing families about available care; the influence of facilitators and local champions on uptake and fidelity during implementation and sustainment; the effects of providing ongoing support to professionals after training; and the role of tailored support in enhancing parental engagement. Parents’ perspectives on the intervention are already being studied in a separate substudy within the larger implementation project,^64^ and preliminary insights have been shared with the professionals in this study. Dynamic adaptations made during the project based on these perspectives will be published separately.^30^ Finally, research on the economic aspects of implementation—such as the costs of training, appointing facilitators and providing refresher courses—is essential to assess long-term feasibility and inform decisions about scaling up.

## Conclusion

This study shows that Soothing and Sleeping is considered a helpful tool for supporting parents with infant crying and sleep problems, when integrated into current YHC practices. We identified Soothing and Sleeping-specific and non-specific implementation barriers and facilitators for YHC, and proposed strategies to enhance future implementation efforts, including proactive outreach to parents, appointing an on-site facilitator, providing ongoing training support for professionals, and tailoring approaches to meet diverse family needs. Addressing identified barriers and investing in facilitators could enhance Soothing and Sleeping adoption, improve infant crying and sleep, strengthen the parent–infant bond and reduce early-life stress.

## Supplementary material

10.1136/bmjpo-2025-003478 online supplemental file 1

## Data Availability

Data is available upon reasonable request.
