# Supplementary material for: Barriers and facilitators perceived by professionals while implementing Soothing and Sleeping in Dutch Healthcare: a mixed-methods study
Source: BMJ Paediatr Open. 2025 Sep 25;9(1):e003478. doi: 10.1136/bmjpo-2025-003478 (PMC12481258; doi:10.1136/bmjpo-2025-003478)
Supplement: online supplemental file 1 [file bmjpo-9-1-s001.docx]

# Appendix A: EBPAS & MIDI questionnaire items

**Questionnaire for professionals – Pre-implementation**

**Section 1 (EBPAS questions)**

**Introduction**The following questions aim to describe your general attitude toward adopting new methods in practice.

**Explanation**The next 8 statements concern your thoughts about using new methods in healthcare, such as new approaches for umbilical granulomas, sleep problems, vision issues, toilet training, etc.

This section does not focus specifically on Soothing and Sleeping but on any possible new method.

Please select the answer that best indicates the degree to which you agree with each statement (1: not at all – 5: to a very large extent):

1. I enjoy applying a new method in healthcare to help my clients.
2. I am willing to try new methods in healthcare, even if it requires me to follow a protocol or guideline.
3. I know better how to care for clients than scientific researchers.
4. I am willing to apply a new method developed by scientific researchers in healthcare.
5. A new healthcare method based on scientific research is not practical in everyday work.
6. Practical experience is more important than using guidelines or protocols.
7. I would not use guidelines or protocols.
8. I would try a new healthcare method, even if it differs significantly from what I am used to doing.

**Explanation**

For the following questions, imagine you received training on a new healthcare method (e.g., for toilet training issues). Select the answer that best indicates how likely you are to apply this method (1: no chance – 5: very high chance).

**How likely are you to apply the method if...**

1. It feels appealing to you?
2. It seems meaningful to you?
3. It is required by your supervisor?
4. It is required by your organization?
5. It is mandated nationally?
6. It is used by colleagues who are satisfied with it?
7. You feel like you received sufficient training to apply it correctly?

**Section 2 (selected MIDI questions)**

**Introduction**
The following questions are specifically about Soothing and Sleeping, based on the Happiest Baby-method by dr. Harvey Karp.

**Explanation**

Please indicate to what extent you agree with the following statement (1: strongly disagree – 5: strongly agree).

1. I consider it part of my role to provide care to parents in the areas of crying, soothing, and sleeping.
2. The method seems suitable for my clients (parents with babies up to 4 months with questions about crying, soothing, and sleeping).
3. Clients (parents and babies up to 4 months with questions about crying, soothing, and sleeping) will generally be satisfied if the method is offered by my organization.

**Explanation**

Please indicate to what extent the following individuals expect you to offer Soothing and Sleeping (1: definitely not – 5: definitely yes).

1. Your direct supervisor/management.
2. Your colleagues within your department, namely the nurses.
3. Your colleagues within your department, namely the medical doctors.
4. Your clients: parents.

**Explanation**

Please indicate how much you take the opinion of the following individuals into account when it comes to working with Soothing and Sleeping (0: very little – 5: very much).

1. Your direct supervisor/management.
2. Your colleagues within your department, namely the nurses.
3. Your colleagues within your department, namely the medical doctors.
4. Your clients: parents.

**Question**

Aside from the introduction of Soothing and Sleeping, are there other changes you are currently dealing with or expect to face soon (reorganization, merger, budget cuts, staff turnover, other innovations)? (indicate yes or no)

**Questionnaire for professionals – Post-implementation**

**Section 1 (EBPAS questions)**

**Introduction**The following questions aim to describe your general attitude toward adopting new methods in practice.

**Explanation**The next 8 statements concern your thoughts about using new methods in healthcare, such as new approaches for umbilical granulomas, sleep problems, vision issues, toilet training, etc.

This section does not focus specifically on Soothing and Sleeping but on any possible new method.

Please select the answer that best indicates the degree to which you agree with each statement (1: not at all – 5: to a very large extent):

1. I enjoy applying a new method in healthcare to help my clients.
2. I am willing to try new methods in healthcare, even if it requires me to follow a protocol or guideline.
3. I know better how to care for clients than scientific researchers.
4. I am willing to apply a new method developed by scientific researchers in healthcare.
5. A new healthcare method based on scientific research is not practical in everyday work.
6. Practical experience is more important than using guidelines or protocols.
7. I would not use guidelines or protocols.
8. I would try a new healthcare method, even if it differs significantly from what I am used to doing.

**Explanation**

For the following questions, imagine you received training on a new healthcare method (e.g., for toilet training issues). Select the answer that best indicates how likely you are to apply this method (1: no chance – 5: very high chance).

**How likely are you to apply the method if...**

1. It feels appealing to you?
2. It seems meaningful to you?
3. It is required by your supervisor?
4. It is required by your organization?
5. It is mandated nationally?
6. It is used by colleagues who are satisfied with it?
7. You feel like you received sufficient training to apply it correctly?

**Section 2 (selected MIDI questions)**

**Introduction**
The following questions are specifically about Soothing and Sleeping, based on the Happiest Baby-method by dr. Harvey Karp.

**Explanation**

Please indicate to what extent you agree with the following statement (1: strongly disagree – 5: strongly agree).

1. The training on using Soothing and Sleeping in Youth Healthcare clearly indicated which activities I should perform and in what order.
2. Soothing and Sleeping is based on factually accurate knowledge.
3. Soothing and Sleeping is too complicated for me to use.
4. Soothing and Sleeping aligns well with how I am used to working.
5. I find the effects of using Soothing and Sleeping clearly visible.
6. I find Soothing and Sleeping suitable for my clients (parents with babies up to 4 months).
7. I believe it is part of my role to provide parents with care in the areas of crying, comforting, and sleeping.
8. Clients (parents with babies up to 4 months) were and will generally be satisfied if Soothing and Sleeping is offered by my organization.
9. Clients (parents with babies up to 4 months) will generally actively listen and want to practice when I explain or demonstrate Soothing and Sleeping.
10. I can rely on sufficient help from my colleagues if I need it when using Soothing and Sleeping.
11. I can rely on sufficient help from my supervisor if I need it when using Soothing and Sleeping.
12. I can rely on sufficient help from my management if I need it when using Soothing and Sleeping.
13. In my organization, measures have been taken to ensure that employees who use Soothing and Sleeping and leave the organization are replaced in time by (new) employees who are sufficiently trained in Soothing and Sleeping.
14. There is enough staff in our organization to use Soothing and Sleeping as intended.
15. Our organization provides me with enough time to integrate Soothing and Sleeping as intended into my daily work.
16. Our organization provides me with enough materials and resources to use Soothing and Sleeping as intended.

**Explanation**

Please indicate for how many of your colleagues this applies, according to you (1: no colleague – 7: all colleagues).

1. Which portion of your colleagues, for whom Soothing and Sleeping is intended, actually use it?

**Explanation**

Please indicate to what extent the following individuals expect you to offer Soothing and Sleeping (1: definitely not – 5: definitely yes).

1. Your direct supervisor/management.
2. Your colleagues within your department, namely the nurses.
3. Your colleagues within your department, namely the medical doctors.
4. Your clients: parents.

**Explanation**

Please indicate how much you take the opinion of the following individuals into account when it comes to working with Soothing and Sleeping (1: very little – 5: very much).

1. Your direct supervisor/management.
2. Your colleagues within your department, namely the nurses.
3. Your colleagues within your department, namely the medical doctors.
4. Your clients: parents.

**Explanation**

The following questions are about how capable you feel in explaining the different parts of the method to parents. For each part, select to what extent this applies to you (1: definitely not – 5: definitely yes).

1. Do you think you would be able to….
2. explain the theory of the fourth trimester to parents?
3. explain swaddling to parents?
4. explain the side/stomach position to parents?
5. explain shushing to parents?
6. explain swinging to parents?
7. explain the use of the pacifier at the right moment during comforting to parents?

**Question**

Aside from the introduction of Soothing and Sleeping, are there other changes you are currently dealing with or expect to face soon (reorganization, merger, budget cuts, staff turnover, other innovations)? (indicate yes or no)

**Explanation**

Here are a number of statements about potential benefits that (the use of) Soothing and Sleeping could bring you personally. Choose the answer that indicates the extent to which you agree with each statement (1: strongly disagree – 5: strongly agree)

1. I feel more confident when parents have questions about crying.
2. I feel more confident when parents have questions about sleeping.
3. I can also practically support my own family and friends with questions about comforting and sleeping.
4. Parents rate my professionalism higher.
5. I experience a better relationship with parents.
6. I am prouder of my profession.
7. I feel more recognition as a professional, because I have the impression that colleagues refer to me more often when there are questions about crying.
8. I feel more recognition because other disciplines (such as maternity care, midwives, and doctors) seem to refer to me more often.
9. I like that I can involve fathers/partners more in comforting and sleeping.
10. I see another potential benefit here, namely… [open field]

**Explanation**

Here are a number of statements about potential disadvantages that (the use of) Soothing and Sleeping could bring you personally. Choose the answer that indicates the extent to which you agree with each statement (1: strongly disagree – 5: strongly agree)

1. Our team is now providing information to parents about crying, comforting, and sleeping that I do not agree with.
2. It goes against my principles to recommend swaddling materials or workshops that parents have to pay for.
3. Because I have to explain the method, I now run out of time during my home visits and consultations.
4. I am unable to finish my work now because everyone refers to me when there are questions about crying and sleeping.
5. I now overestimate my own expertise and use knowledge from partners too late.
6. I am more likely to give family and friends unsolicited advice about sleeping and crying.
7. I am afraid that my family and friends may find me strict or judgmental when I explain Soothing and Sleeping to them.
8. I see another potential disadvantage here, namely… [open field]

**Explanation**

Here are a number of goals that could be achieved with families through the use of Soothing and Sleeping. Choose the answer that indicates the extent to which you agree with each statement (1: definitely not – 5: definitely yes).

I expect that with Soothing and Sleeping, the following goals for families will be achieved…

1. That parents receive tailored care for questions about crying and sleeping.
2. That both fathers and mothers can comfort their baby.
3. That parents can follow practical advice for dealing with their baby's sleep problems.
4. That parents feel more confident in responding sensitively to their baby.
5. That parents learn more about baby care without having to read books.

# Appendix B: Topic guides for focus groups with professionals

**Focus Group Professionals – Pre-implementation**

**Question 1**
How does the intervention Soothing and Sleeping compare to other - similar or alternative - interventions within your organization?
*Please also consider the ways in which these are offered.*

And only if applicable:

- What are the advantages of the care offering Soothing and Sleeping compared to other interventions?
- What are the disadvantages of the care offering Soothing and Sleeping compared to other interventions?

**Question 2**
In your opinion, what are the barriers and facilitators for an optimal implementation of the intervention Soothing and Sleeping within your organization?
*By optimal implementation, we mean that the care offering is introduced/integrated in a process-oriented and planned way that suits both the providers (you) and the users (parents in vulnerable circumstances).*

**Follow-up questions:**

- **For the providers:** What would or wouldn’t work well for you? What would or wouldn’t motivate you? In other words, what are your needs, preferences, and wishes for being able to (continue to) offer the care during the pilot? And why?
- **For the users:** What would or wouldn’t work well for parents? What would or wouldn’t motivate them? In other words, what are the needs, preferences, and wishes of parents for being able to (properly) use the care during the pilot implementation? And why? Consider both the workshop and the one-on-one guidance.

Rank the answers to Question 2 (identify critical determinants).

**Additional question for the national sample only:**

What outcome measures would be decisive for you in choosing to use Soothing and Sleeping in your organization? And to whom (which stakeholder group) do these outcomes pertain?

**Focus Group Professionals – Post-implementation**

**1. Fidelity**

Perceptions of the extent to which JGZ professionals feel capable of offering the method according to the protocol.

**Questions**

- Do you apply Soothing and Sleeping in your daily work? How often, in what ways? How much time did the explanation take (in the consultation office, during home visits)?
- Do you now actively ask about crying and sleeping during the first three contact moments with parents?
- Which part of the training was most useful/used by you? Do you feel you received sufficient training to apply the method faithfully in workshops or individual consultations? Did you miss anything in the training? What was unnecessary?
- Have you learned from or supported each other in using Soothing and Sleeping? Was this during case discussions, team meetings, or at another moment?

1. **Feasibility**

Perception of the extent to which Soothing and Sleeping can be successfully offered at the implementation sites, and the barriers and facilitators influencing this.

**Questions**

- Did you encounter specific challenges when using the method that hindered the use of Soothing and Sleeping? And what facilitated its use?
- Which materials do you use to offer the method? Are there any materials you are missing?
- What do you envision for the future of Soothing and Sleeping in your area?
- Optional follow-up: Are you the best organization to implement Soothing and Sleeping in your district? Why or why not? What about partnerships with other stakeholders?

**3. Appropriateness**

Perception of the suitability, relevance, and compatibility of Soothing and Sleeping for the implementation site, care providers, and clients.

**Questions**

- Have you come across any cases where Soothing and Sleeping may not have been a good fit?
- Follow-up: Did you reach vulnerable parents and did you actively offer the method during pregnancy or after birth? Was this via consultations/home visits or workshops? What aspects of the method did they use? Did you need to adjust your explanation?

**Additional Focus Group with Workshop Leaders – Post-implementation**

**Title/Content of the Workshop**

- Which workshop content helped parents most, and how did you notice this? Why?
  - Do you discuss content in the same way in consultations as in workshops?
  - Did parents raise questions or did you come across topics that were missing from the PowerPoint?
  - Were there any topics left undiscussed in workshops that you would like to add about crying, comforting, or sleeping?
- Does the title/content of the workshop align with how you present it? Did parents share whether the workshop met their expectations?

**Workshop Experiences**

- You all received SOOTHING AND SLEEPING training, including theoretical information and practice. Have you ever forgotten parts of the theory or method during a workshop? How did you handle that?
  - Where do you search for any missing/forgotten information?
  - Do you ever feel uncertain in such situations? How do you deal with it?
  - Do you feel confident conducting workshops, and why (not)?
    - Can you provide examples of when you felt confident or not? What do you need to feel more confident?
- How was practicing swaddling for you?
  - How did you manage workshops with multiple families?
  - How was it to hold parents’ babies or practice with dolls?
  - How did you handle not being able to assist everyone at once during swaddling practice?
  - What do you think of using blankets for swaddling? And swaddle sacks? What do you usually recommend to parents?
- What did you think of the combination of one more extensively trained professional and one less trained professional per workshop?
- How much time did each workshop take (including preparation)? Was 4 hours per session sufficient/too much/too little? How much time was needed for preparation/closure?
- What does a workshop location need for you to feel like you can provide effective group education to parents in your are? Why? Can you provide an example?
- What are your thoughts on offering workshops in multiple languages?
- What if a parent at your consultation speaks a different language? Can you share examples of how you handled this?
- How did the workshops align with other care for young parents in the area (e.g., weigh-ins, Baby’s Best Start, baby massage, mom cafés)?

**Future Workshops**

- How do you feel about continuing the workshops?
- Initially, time constraints seemed problematic, but there now appears to be enthusiasm for continuation. Why is it working now?
- Some suggested a drop-in format might be better than workshops. Would you like to elaborate? What would that look like?
- What excites you about using Soothing and Sleeping in your area in the future?

# Appendix C: Implementation procedure

Implementation of the Happiest Baby-method into the implementation sites consisted of the following two steps.

*Step 1*

Teams at the implementation sites received training in Soothing and Sleeping, consisting of preparatory reading about Soothing and Sleeping’s theory and practical applications, and a two-hour team training session with an Soothing and Sleeping-certified educator (MHa). This training prepared professionals to educate parents individually on Soothing and Sleeping’s theory and to practice soothing techniques with them. Additionally, two nurses per team underwent further training by certified Soothing and Sleeping educators (EM, HRR, and MHa) to lead parental workshops. This training included reading of two books and 26 scientific articles, five hours of individual theoretical instruction, and six hours of coaching on workshop facilitation. After observing a workshop and gradually taking over the instructor role over two sessions, those nurses completed an examination with 55 multiple-choice questions and received the Happiest Baby Educator certificate (Happiest Baby, 2023).

*Step 2*

Team members were expected to apply their trained skills by guiding parents individually, while certified nurses additionally hosted monthly Soothing and Sleeping workshops. A Soothing and Sleeping workshop accommodated up to six families (one or two caregivers, pregnant or with their 0-4 month old baby) and included a presentation on Soothing and Sleeping theory, skills training on the five S’s, and practice time with the baby or a doll. Participation costs were 25 euros, or 5 euros for low-income families. The workshop could be offered free-of-charge to families in vulnerable situations to facilitate participation. Parents received a swaddle blanket and an informational booklet including the five steps for home review. Parents who had individual guidance received a handout.

Happiest Baby I (2023) *Happiest Baby Educator Certification Program*. Available at: <https://thehappiestbaby.org/> (accessed October 17th, 2023).

# Appendix D: Reflexivity

To enhance transparency and credibility, we reflected on how our professional and personal backgrounds may have shaped data collection, facilitation, and interpretation. JvS, an MSc in Public Health with no prior involvement in Soothing and Sleeping, contributed methodological oversight and emphasized systematic coding and analytic rigor. EM, a senior researcher and orthopedagogue with extensive experience in child development and in evaluating Soothing and Sleeping, co-facilitated one focus group with JvS. Her expertise may have informed the framing of questions and interpretation, particularly regarding intervention effectiveness. HR, also a senior researcher and orthopedagogue, has both researched and practiced Soothing and Sleeping and was also involved in training professionals in the intervention; while she was only marginally engaged in the interpretation process, her dual role as researcher and trainer could have shaped perspectives on how professionals described their experiences. AP, a PhD candidate and Youth Health Care physician, contributed clinical insights on infant sleep and healthcare practice, which may have heightened sensitivity to feasibility and applicability in daily care. She was mainly involved in project group meetings where preliminary data were presented. MHo, a senior researcher and coordinator of the Academische Werkplaats Jeugd en Gezondheid, drew on her long-standing collaboration with youth healthcare organizations, which may have sensitized her to organizational barriers and facilitators. She did not have prior experience with Soothing and Sleeping, and was mainly involved in project group meetings where preliminary data were presented. Finally, MHa, a Youth Health Care physician and researcher, also trained professionals in Soothing and Sleeping and was involved in data interpretation; her dual role may have directed attention to the practical usability and adoption of the method. By explicitly acknowledging these positionalities, we sought to remain aware of possible biases while also using our diverse expertise to enrich the depth and trustworthiness of the findings.
