# Supplementary material for: Barriers and facilitators perceived by professionals while implementing Soothing and Sleeping in Dutch Healthcare: a mixed-methods study
Source: BMJ Paediatr Open. 2025 Sep 25;9(1):e003478. doi: 10.1136/bmjpo-2025-003478 (PMC12481258; doi:10.1136/bmjpo-2025-003478)
Supplement: Supplementary file 2 [file bmjpo-2025-003478.reviewer_comments.pdf]

PEER REVIEW HISTORY

BMJ Paediatrics Open publishes all reviews undertaken for accepted manuscripts. Reviewers are asked to complete a checklist review form and are provided with free text boxes to elaborate on their assessment. These free text comments are reproduced below.

ARTICLE DETAILS

**TITLE (PROVISIONAL)**	Barriers and facilitators perceived by professionals while implementing Soothing and Sleeping in Dutch Healthcare: a mixed-methods study
**AUTHORS**	van Someren, Jamie; Möller, Eline; Rodenburg, Hesther; Peters, Annemieke; Hoogsteder, Mariëtte; Harskamp-van Ginkel, Margreet

VERSION 1 - REVIEW

**REVIEWER NAME**	Harriet Hiscock
**REVIEWER AFFILIATION**	Royal Children's Hospital, Centre for Community Child Health
**REVIEWER CONFLICT OF INTEREST**	N/A
**AI DISCLOSURE**	No
**DATE REVIEW RETURNED**	02-Aug-2025

**GENERAL COMMENTS**	Introduction<br>– you state that the 5s's 'activate the infant's calming response by mimicking soothing womb-like rhythms, thereby promoting self-regulation and sleep (4, 5, 7).' But what evidence is there that this is the underlying mechanism at a biological/physiological level? It may be better to say that this MAY activate the infant's calming response.<br><br>Methods<br>- on page 6, define what you mean by 'vulnerable situations '- is this domestic violence, maternal mental health problems, other?<br>- The Dynamic Adaptation Process (DAP) Framework also include clients – make clear in the Methods that you are not reporting on clients' own views or perceptions in this paper<br>- Role of CALD in acceptance of the Soothing and Sleeping intervention – was the YHC's professional's cultural/linguistic background measured the YHC pre-implementation questionnaire?<br>- how did you recruit the YHC professionals- snowballing? purposive sampling? Other? (see COREQ checklist - https://www.equator-network.org/reporting-guidelines/coreq/ as an example of a checklist on reporting qualitative studies)<br>- were particiapnts given a chance to feedback on the study findings? This is best practice in qual research and if they did not get a chance, mention this as a limitation.<br><br>- What is/are the professional background of the FG facilitators? Can you reflect on how their background may have affected interpretation of the qualitative findings?<br>- In your pre-post quantitative analyses, what statistical tests did you use for the MIDI and EBPAS-15? If you did not do testing for statistical significance, say why (e.g. small sample size, exploratory analysis only) Also, say how you scored (total score?) the measures and if there are any norms against which to compare your sample.

	Results<br>- What was your response rate for the YHC professionals?<br>- Can you make clear why the organisations charged families to attend workshops of the Soothing and Sleeping intervention? Is there a cost to running/licensing this program? If yes, you should include this in your Methods.<br>- Did YHC staff need to rule out medical causes of infant crying (e.g. food allergies) before implementing the program? If not, could the intervention cause indirect harm by not treating underlying medical causes of crying?<br>- For the EBPAS – are there any norms? Were the pre-implementation scores already high and this is why you did not see much change? Does this also apply to the MIDI? Some clinical interpretation of the scores and the change in scores is needed.<br>Discussion<br>- this is well written but an additional limitation could be not knowing the cultural/linguistic background of the professionals and whether this may influence their uptake of the intervention<br>- Include a section on Future Research - e.g. to understand clients' own views and perceptions of the intervention within the YHC services; document the costs of implementing and maintaining the intervention (given you have suggested facilitators and refresher courses)

VERSION 1 – AUTHOR RESPONSE

Reviewer Comments and Responses

Introduction

Comment 1: You state that the 5s's 'activate the infant's calming response by mimicking soothing womb-like rhythms, thereby promoting self-regulation and sleep (4, 5, 7).' But what evidence is there that this is the underlying mechanism at a biological/physiological level? It may be better to say that this MAY activate the infant's calming response.

Response 1: We agree with the reviewer's point and have revised the statement to be more cautious, noting that the 5s's may activate the infant's calming response.

Edit 1: "These steps may activate the infant's calming response by mimicking soothing womb-like rhythms, thereby promoting self-regulation and sleep (4, 5, 7)."

Methods

Comment 2: On page 6, define what you mean by 'vulnerable situations '- is this domestic violence, maternal mental health problems, other?

Response 2: Thank you for your suggestion. We have clarified the definition of “vulnerable situations” in the study design section.

Edit 2: “Vulnerability in a family was defined as having a low income (defined as <150% minimum income), parental unfamiliarity with the Dutch healthcare system, or long-term parental mental/cognitive vulnerabilities (i.e. anxiety disorder, depression, AD(H)D, autism, low literacy, mild intellectual disabilities, or substance abuse).”

Comment 3:

The Dynamic Adaptation Process (DAP) Framework also include clients – make clear in the Methods that you are not reporting on clients’ own views or perceptions in this paper.

Response 3: We have added a sentence to the method’s qualitative analysis section clarifying that our focus is on professionals’ perspectives, not clients’ own views.

Edit 3: “Regarding the DAP framework, our focus for this paper is on professionals' views on the four levels, and we are not reporting on clients’ own views or perceptions.”

Comment 4: Role of CALD in acceptance of the Soothing and Sleeping intervention – was the YHC’s professional’s cultural/linguistic background measured the YHC pre-implementation questionnaire?

Response 4: We did not measure professionals’ cultural/linguistic background. We have acknowledged it as a limitation in the Discussion as we agree that this could potentially influence their acceptance of Soothing and Sleeping. From what we know about the participants ourselves, most participants were Dutch, and some were second generation immigrants from non-western countries (e.g., Turkey, Marocco, Suriname). All professionals had Dutch as their native language, but speak two or more languages fluently.

Edit 4: “Another limitation is that the cultural and linguistic backgrounds of professionals were not assessed, although these factors may influence their acceptance of interventions such as Soothing and Sleeping, particularly components like swaddling. Swaddling is considered a common and beneficial soothing practice in some cultural contexts, whereas in others it may be discouraged due to concerns about infant safety or developmental outcomes (62). Furthermore, differences in language and culture between professionals and families may affect how confidently recommendations are communicated and received (63). Such barriers could potentially influence both the uptake by professionals, as well as by parents. However, our MIDI measures indicated that professionals considered Soothing and Sleeping relevant for parents, and 81% integrated Soothing and Sleeping into their daily practice, with professionals not implementing it providing only reasons such as not working with the target group or not encountering a need to implement it.”

Comment 5: How did you recruit the YHC professionals- snowballing? purposive sampling? Other? (see COREQ checklist - https://www.equator-network.org/reporting-guidelines/coreq/ as an example of a checklist on reporting qualitative studies)

Response 5: Thank you for the clarifying question. We have clarified the recruitment approach of YHC professionals in the Methods section.

Edit 5: “All YHC professionals (youth nurses, medical doctors, policymakers, and social workers) working at the implementation sites were recruited via their supervisors, while the NCJ invited national participants purposively based on expertise, location, and role.”

Comment 6: Were participants given a chance to feedback on the study findings? This is best practice in qual research and if they did not get a chance, mention this as a limitation.

Response 6: Thank you for pointing this out, our participants were provided the opportunity to provide feedback on the findings, we have clarified this in the Methods section.

Edit 6: “To further enhance credibility, participants were invited to review and provide feedback on the preliminary findings during two separate meetings. These findings were also presented to a broader audience at two annual local meetings attended by professionals, researchers, and policy makers involved in YHC. In addition, preliminary results were regularly discussed in project group meetings comprising one participant, researchers, and policy makers. All feedback was considered during interpretation of the results.”

Comment 7: What is/are the professional background of the FG facilitators? Can you reflect on how their background may have affected interpretation of the qualitative findings?

Response 7: We have added information on the fact that the facilitators’ professional background may influence interpretation of the findings in the Methods, and referred the reader to the appendices to read a reflection on the influence of our backgrounds.

Edit 7: In the main-text: “To enhance transparency and credibility, we reflected on how the professional and personal backgrounds of the research team may have influenced data collection, facilitation, and interpretation in Appendix D.”

In Appendix D: “To enhance transparency and credibility, we reflected on how our professional and personal backgrounds may have shaped data collection, facilitation, and interpretation. JvS, an MSc

in Public Health with no prior involvement in Soothing and Sleeping, contributed methodological oversight and emphasized systematic coding and analytic rigor. EM, a senior researcher and orthopedagogue with extensive experience in child development and in evaluating Soothing and Sleeping, co-facilitated one focus group with JvS. Her expertise may have informed the framing of questions and interpretation, particularly regarding intervention effectiveness. HR, also a senior researcher and orthopedagogue, has both researched and practiced Soothing and Sleeping and was also involved in training professionals in the intervention; while she was only marginally engaged in the interpretation process, her dual role as researcher and trainer could have shaped perspectives on how professionals described their experiences. AP, a PhD candidate and Youth Health Care physician, contributed clinical insights on infant sleep and healthcare practice, which may have heightened sensitivity to feasibility and applicability in daily care. She was mainly involved in project group meetings where preliminary data were presented. MHo, a senior researcher and coordinator of the Academische Werkplaats Jeugd en Gezondheid, drew on her long-standing collaboration with youth healthcare organizations, which may have sensitized her to organizational barriers and facilitators. She did not have prior experience with Soothing and Sleeping, and was mainly involved in project group meetings where preliminary data were presented. Finally, MHa, a Youth Health Care physician and researcher, also trained professionals in Soothing and Sleeping and was involved in data interpretation; her dual role may have directed attention to the practical usability and adoption of the method. By explicitly acknowledging these positionalities, we sought to remain aware of possible biases while also using our diverse expertise to enrich the depth and trustworthiness of the findings."

Comment 8: In your pre-post quantitative analyses, what statistical tests did you use for the MIDI and EBPAS-15? If you did not do testing for statistical significance, say why (e.g. small sample size, exploratory analysis only). Also, say how you scored (total score?) the measures and if there are any norms against which to compare your sample.

Response 8: We have clarified the statistical approach for the MIDI and EBPAS-15 in the Methods, explained the scoring, and noted that analyses were exploratory given the small sample size. We have also acknowledged the lack of norm comparisons where applicable. The only norm that we were able to identify were normative total mean EBPAS-15 scores for mental health providers in the United States, which typically ranged around $M \approx 2.9–3.3$ (depending on subscale and provider characteristics). The pre-implementation mean found in our study ($M = 3.04$) is close to their norm, whereas the post-implementation mean ($M = 3.64$) for our study is above the typical U.S. norms. However, since there may be major differences in professional training, service context, and cultural attitudes toward interventions between U.S. mental health providers and Dutch Youth Healthcare providers, we have decided not to compare the two in our analysis. Moreover, since we have a small sample size, we want to be cautious in making any statistical claims. For the MIDI, we have not found any norms to compare our sample to.

Edit 8: "Pre- and post-implementation responses were compared based on mean scores, to assess attitude shifts attributable to study participation. We did not test for statistical significance, due to the small sample size and exploratory nature of the quantitative analysis, neither did we identify any norms suitable to compare our EBPAS-15 or MIDI scores to."

Results

Comment 9: What was your response rate for the YHC professionals?

Response 9: For the national sample, the response rate is unknown due to recruitment by an external party. For the implementation sites, all invited team members participated pre-implementation. This is now clarified in the Results section. In the original manuscript there was a footnote included in Table 1 to explain participation throughout the implementation process, this has now been moved to the text to provide more clarification.

Edit 9: "In total, 41 YHC professionals participated in this study, including 16 from the national sample and 25 from the implementation sites. For the implementation sites, all invited team members participated pre-implementation, but participation throughout the implementation process was influenced by staff turnover, sickness, and maternity leave. Therefore, 12 participants contributed across all phases, 7 participated only before and during implementation, and 6 during and post-implementation (they were included at a later stage). For the national sample, 16 participants were selected and agreed to participate from a group of 40 active members of a YHC science consultant group.

Comment 10: Can you make clear why the organisations charged families to attend workshops of the Soothing and Sleeping intervention? Is there a cost to running/licensing this program? If yes, you should include this in your Methods.

Response 10: We have clarified the reason families were charged in the Methods.

Edit 10: "For parental workshops, families were charged €5 if they had a low income and €25 otherwise. The fee covered the provision of a swaddle blanket and an informational booklet for home use. The fee also helped, based on the organization's prior experience, to reduce non-attendance, as free registration was associated with higher rates of no-shows."

Comment 11: Did YHC staff need to rule out medical causes of infant crying (e.g. food allergies) before implementing the program? If not, could the intervention cause indirect harm by not treating underlying medical causes of crying?

Response 11: In the Dutch Youth healthcare system, all children are monitored regularly from 2 weeks of birth onwards by YHC professional (e.g., YHC nurses and medical doctors). Medical causes for infant crying were investigated during all periodic health check-ups and demand-driven, tailor-made care appointments. If infant crying did not have a medical cause or persisted after medical treatment, Soothing and Sleeping was advised to parents. In practice, we noticed that even in workshops, parents could express any medical concerns and the nurses either answered their questions immediately or planned a follow-up appointment with parents. We have clarified the role of YHC staff in identifying medical causes of infant crying before implementation in the Introduction.

Edit 11: “They can provide adaptive extra support where indicated, for example ruling out underlying medical causes for infant crying and implementing Soothing and Sleeping.”

Comment 12: For the EBPAS – are there any norms? Were the pre-implementation scores already high and this is why you did not see much change? Does this also apply to the MIDI? Some clinical interpretation of the scores and the change in scores is needed.

Response 12: Thank you for the clarifying question. In the Methods section we now state that we did not identify any norms. We have added a section to the Discussion providing clinical interpretation of the EBPAS and MIDI scores, including the possibility that high baseline values limited observed change.

Edit 12:

In the methods section: “Pre- and post-implementation responses were compared based on mean scores, to assess attitude shifts attributable to study participation. We did not test for statistical significance, due to the small sample size and exploratory nature of the quantitative analysis, neither did we identify any norms suitable to compare our EBPAS-15 or MIDI scores to.”

In the discussion section: “For the EBPAS, the relatively high scores pre-implementation may partly explain why only limited change was observed post-implementation. Nevertheless, the modest increase at one site suggests a further strengthening of positive attitudes after implementing Soothing and Sleeping, while stable scores at the second site suggest sustained receptivity. For the MIDI, the fact that professionals already perceived Soothing and Sleeping as relevant and expected high client satisfaction may also explain the lack of measurable change post-implementation. However, the slight increase in reported influence from colleagues, supervisors, and parents suggests that organizational and social dynamics became more important during implementation. Moreover, the high rate of integration into practice (81%) underscores the intervention’s feasibility and acceptability in YHC.”

Discussion

Comment 13: This is well written but an additional limitation could be not knowing the cultural/linguistic background of the professionals and whether this may influence their uptake of the intervention.

Response: Thank you for your suggestion. We have added this point as an additional limitation in the Discussion.

Edit 13: “Another limitation is that the cultural and linguistic backgrounds of professionals were not assessed, although these factors may influence their acceptance of interventions such as Soothing and Sleeping, particularly components like swaddling. Swaddling is considered a common and beneficial soothing practice in some cultural contexts, whereas in others it may be discouraged due to concerns about infant safety or developmental outcomes (62). Furthermore, differences in language and culture between professionals and families may affect how confidently recommendations are communicated and received (63). Such barriers could potentially influence both the uptake by professionals, as well as by parents. However, our MIDI measures indicated that professionals considered Soothing and Sleeping relevant for parents, and 81% integrated Soothing and Sleeping into their daily practice, with professionals not implementing it providing only reasons such as not working with the target group or not encountering a need to implement it.”

Comment 14: Include a section on Future Research - e.g. to understand clients’ own views and perceptions of the intervention within the YHC services; document the costs of implementing and maintaining the intervention (given you have suggested facilitators and refresher courses).

Response 14: Thank you for the suggestion. We have added a short section on Future Research in the Discussion highlighting the need for studies on clients’ perspectives and the costs of implementing and maintaining the intervention, as well as the need to evaluate the recommended implementations strategies.

Edit 14: “When implementation strategies are used, we recommend process evaluation to investigate fidelity and sustainability of Soothing and Sleeping across different care settings. Key areas for evaluation include: the impact of actively informing families about available care; the influence of facilitators and local champions on uptake and fidelity during implementation and sustainment; the effects of providing ongoing support to professionals after training; and the role of tailored support in enhancing parental engagement. Parents’ perspectives on the intervention are already being studied in a separate sub-study within the larger implementation project (64), and preliminary insights have been shared with the professionals in this study. Dynamic adaptations made during the project based on these perspectives will be published separately (30). Finally, research on the economic aspects of implementation—such as the costs of training, appointing facilitators, and providing refresher courses—is essential to assess long-term feasibility and inform decisions about scaling up.”
